# A Novel Mutation in the Androgen Receptor Gene of Female Patients with 46,XY Karyotype

**DOI:** 10.3390/cimb47050349

**Published:** 2025-05-10

**Authors:** Inayet Nur Uslu, Nuriye Gokce, Gulsevinc Aksoy, Nihal Inandiklioglu, Bilgin Yuksel, Munis Dundar, Osman Demirhan

**Affiliations:** 1 Department of Medical Biology, Faculty of Medicine, Çukurova University, 01330 Adana, Türkiye; inuruslu@gmail.com (I.N.U.); gulsevinc91ay@gmail.com (G.A.); 2 Department of Medical Genetics, Faculty of Medicine, Erciyes University, 38280 Kayseri, Türkiye; nuriyecoskun@erciyes.edu.tr (N.G.); dundar@erciyes.edu.tr (M.D.); 3 Department of Medical Biology, Faculty of Medicine, Yozgat Bozok University, 66100 Yozgat, Türkiye; 4 Department of Pediatrics, Division of Pediatric Endocrinology, Faculty of Medicine, Çukurova University, 01330 Adana, Türkiye; byuksel@cu.edu.tr

**Keywords:** androgen insensitivity syndrome, androgen receptor gene, CAIS, novel mutation

## Abstract

Background: In this study, we aimed to analyze androgen receptor (*AR*) gene mutations in five members of a family with complete androgen insensitivity syndrome (CAIS). Methods: Peripheral blood samples were collected from the proband and four relatives (mother, sister, and two aunts). Cytogenetic imaging and chromosomal analysis were per-formed to elucidate the genetic basis of the condition. Clinical Exome Sequencing (CES) was conducted to identify candidate variants, which were subsequently validated using Sanger sequencing. Evolutionary conservation analysis was performed for the identified *AR* gene mutation. Results: Our analyses revealed that the proband, sister, Aunt I, and Aunt II exhibited a 46,XY karyotype and carried the *SRY* gene. The mother, however, had a 46,XX karyotype, and did not carry the SRY gene, confirming X-linked recessive inheritance of the condition. CES results demonstrated that the proband, sister, Aunt I, and Aunt II harbored a hemizygous c.2246C>T (p.Ala749Val) mutation, while the mother carried this mutation in a heterozygous state. The presence of this mutation was confirmed by Sanger sequencing. Evolutionary conservation analysis indicated that the mutation is conserved among vertebrates. Conclusion: in conclusion, we identified a novel missense mutation (c.2246C>T) in the *AR* gene in five members of a CAIS-affected family, which has not been previously reported in the literature.

## 1. Introduction

The genetic mechanism underlying sex determination and differentiation is governed by an extremely complex process. In humans, the typical male and female sexes are primarily determined by the presence of XX and XY karyotypes, respectively [1]. Androgens play a critical role in sex differentiation and the development of male sexual characteristics. In humans, androgens exert their function by forming complexes with the androgen receptor (AR) [2]. The *AR* is essential for male sexual differentiation, as it is a ligand-activated transcription factor that is activated upon binding to testosterone and dihydrotestosterone [3].

Disorders of Sex Development (DSD) are congenital conditions characterized by abnormalities in chromosomal, gonadal, or phenotypic sex. Based on 2006 Chicago classification, DSDs are divided into three categories: sex chromosome DSD, 46,XY DSD, and 46,XX DSD. Sex chromosome DSDs include Klinefelter syndrome, Turner syndrome and mosaics with 45,X/46,XY or 46,XX/46,XY. These classes can be divided into subheadings [4]. The 46,XY DSDs include disorders of gonadal development, disorders of androgen synthesis, disorders of androgen action, persistent Müllerian duct syndrome, and unclassified disorders. The 46,XX DSDs include disorders of gonadal development, disorders of androgen excess and unclassified disorders [5]. However, most DSD phenotypes are rare. Among these, androgen insensitivity syndrome (AIS) represents the most common cause of 46,XY DSD [6]. The prevalence of AIS in males has been reported to range between 1 in 20,400 and 1 in 99,100 [7]. AIS manifests in three distinct phenotypic forms: complete AIS (CAIS), characterized by a normal female phenotype with external genitalia; partial AIS (PAIS), associated with ambiguous genitalia, hypospadias, or micro penis; and mild AIS, which presents with a typical male phenotype but infertility [8]. Individuals with CAIS exhibit female external genitalia and the absence of male reproductive structures such as the epididymis, vas deferens, seminal vesicles, and prostate, as well as gynecomastia and a lack of pubic and axillary hair [9]. Typically, CAIS patients present with a clearly normal female phenotype at birth. They lack a cervix and uterus, have a blind-ending vagina, and experience an increase in testosterone levels during puberty, accompanied by elevated luteinizing hormone (LH) levels due to androgen resistance. Furthermore, AIS is associated with an increased risk of testicular cancer in males due to AR gene mutations. CAIS could be associated with increased testicular germ cell tumors incidence compared with the general population. Given the increased risk of testicular germ cell tumors in patients with CAIS, gonadectomy is often recommended alongside puberty induction [8,10].

The primary cause of AIS is androgen insensitivity resulting from mutations in the *AR* gene [11]. The *AR* gene plays a pivotal role in male sex differentiation during fetal development. Located on the X chromosome at Xq11–12, this gene encodes a protein of 919 amino acids, and comprises eight coding exons spanning approximately 90 kb. The AR protein is composed of four functional domains: (1) the *N*-terminal activation domain (NTD, exon 1, residues 1–556), (2) the DNA-binding domain (DBD), (3) the hinge region (HR), and (4) the carboxyl-terminal ligand-binding domain (LBD, residues 669–919). The LBD facilitates ligand binding through a ligand-binding pocket composed of 12 α-helices [12]. Most pathogenic variants in the *AR* gene result in loss of receptor function, leading to peripheral androgen resistance. To date, over 1000 AIS-causing mutations have been identified in this gene [13]. Approximately 90% of these mutations have been linked to AIS, with the majority originating from the maternal germline [14]. However, in some patients, *AR* mutations may arise due to de novo mutations occurring postzygotically. The majority (~two-thirds) of *AR* mutations are inherited from asymptomatic mothers as germline variants, while others result from somatic or de novo mutations. De novo mutations account for approximately 30% of *AR* mutations, whereas the remaining 70% are transmitted in an X-linked recessive manner. Consequently, mothers of affected 46,XY individuals may be heterozygous carriers without displaying any phenotypic abnormalities. In this condition, there is a 50% probability that XY offspring will be affected, while XX offspring will be healthy carriers [7]. A significant proportion of these mutations impair the ligand-binding capacity of the mutant AR protein. Missense mutations are the most frequently observed genetic alterations and are predominantly located within two critical domains of the AR protein. Studies have reported that in CAIS patients, 66.7% of mutations are single-nucleotide missense variants affecting the LBD region. Additionally, frameshift mutations due to deletions and splice-site mutations have also been identified in CAIS [15].

In this study, we aimed to investigate the molecular characterization, phenotypic variations, and genotype-phenotype correlations in a family with 46,XY DSD to elucidate the pathogenesis of CAIS.

## 2. Materials and Methods

This study was performed in line with the principles of the Declaration of Helsinki. Approval was granted by the Ethics Committee of Çukurova University (Adana, Türkiye) (04.01.2024/140-42). In our study, peripheral blood samples were collected from 5 people who are the proband and her relatives (sister, mother and two aunts). Molecular and cytogenetic analyzes were performed.

### 2.1. Clinical Property of the Patients

**Figure 1:**
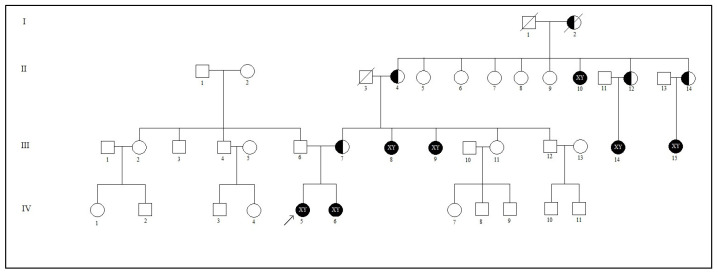
Pedigree of the family with CAIS (the arrow indicates the proband). (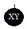: Female phenotype with 46,XY karyotype; 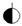: Female who carriers novel mutation).

When the proband (IV.5) was 40 days old, she was admitted to the hospital because of a palpable mass in the inguinal region, and phenotypically had a female appearance. On examination, bilateral inguinal masses consistent with testicular tissue were palpated. In abdominal USG examination, the liver, gallbladder, spleen, pancreas, bladder, and kidneys were normal. However, the uterus and ovaries could not be demonstrated. In bilateral inguinal canals, there were structures of 1.36 × 0.51 cm on the right and 1.37 × 0.45 cm on the left, which were thought to be compatible with the testis. She was sent to the Gender Council when she was 7 months old. The pedigree of the family was drawn based on the information provided by the proband’s mother and aunts. The pedigree is shown in Figure 1.

In our study, the mother, sister, and 2 aunts from the relevant family, apart from the proband, were examined for mutation. It was reported that the proband’s two maternal aunts aged 23 (III.8) and 15 (III.9) also had a similar disease. It was reported that Aunt I (III.8) had inguinal region pain. Since Aunt II (III.9) was attending school, her family chose not to disclose her condition to avoid potential psychological distress. During the physical examination for Aunt I (III.8), it was determined that she was 168 cm tall, weighted 57 kg, had female phenotype, retarded breast development, and scanty pubic and axillary hair growth. Infantile female external genitalia were also observed. But, there was a palpable mass in the inguinal region. Aunt II (III.15) had primary amenorrhea. She had the general appearance of Aunt I (III.8) with CAIS. She had no clinical record of past serious illnesses. No diagnostic procedures had previously been performed to elucidate her disease. During her physical examination, it was determined that she was 172 cm tall, weighted 62 kg, and was female phenotype with sparse pubic and axillary hair. Breast development was bilaterally inadequate, and she had infantile female external genitals. But, there was a palpable mass in the inguinal region, like her sister.

### 2.2. Cytogenetics Analysis

The peripheral blood samples collecting in heparin vacutainers were used for cytogenetic analysis. Blood samples of 300 µL were incubated in RPMI-1640 media (Sigma-Aldrich, St. Louis, MO, USA) at 37 °C for 72 h. The blood cultures were treated with a hypotonic solution (0.075 M KCl), fixed in methanol: acetic acid (3:1). Then, the cell solution was dropped on pre-chilled slides. Giemsa–Trypsin–Giemsa (GTG) banding was applied to the chromosomal preparations and 50 metaphases were examined for each sample. This procedure was carried out at Çukurova University, Department of Medical Biology (Adana, Türkiye).

### 2.3. Molecular Analysis

Genomic DNA samples were extracted from peripheral blood samples of the five patients using the automated system (Zeesan Lab-Aid 824s, Xiamen City, Fujian Province, P.R. China) blood DNA extraction kit. DNA samples were used for detecting the disease-causing mutations and showing SRY region in patients. Molecular Analysis was performed at Erciyes University, Department of Medical Genetics (Kayseri, Türkiye).

### 2.4. QF-PCR Analysis

Multi-STR of chromosomes 13, 18, 21, X, and Y trisomy detection kit (AneuSure, Genetek Biopharma, Berlin, Germany) was used for showing the Short Tandem Repeat (STR) of the SRY gene. The kit includes fluorescently labeled primers for some STR markers in above chromosomes. After genomic DNA samples were extracted from peripheral blood samples, QF-PCR was performed according to the manufacturer’s instructions.

### 2.5. Next-Generation Sequencing (NGS)

NGS was carried out for a CES gene panel containing 6380 genes. We performed targeted exome sequencing at Erciyes University, Department of Medical Genetics (Kayseri, Türkiye) using Sophia Genetics Custom Exome Solution kit (Illumina NextSeq 500 platform, Illumina, Hayward, CA, USA). The detected mutation was classified according to American College of Medical Genetics and Genomics (ACMG) guidelines [16].

### 2.6. Sanger Sequencing Analysis

Sanger sequencing was used to confirm mutations identified in NGS. Briefly, Exon 5 of the *AR* gene was amplified by Polymerase Chain Reaction (PCR) using the following primer sequences; forward primer: 5′-AGCATCTCTGCCCAACAGGGACTCA-3′ and reverse primer: 5′-CCTCATACTGGATTGGCTGGCTGGG-3′. The PCR mix was held 95 °C for 5 min. After that, PCR was set as 30 cycles, and each cycle was applied as follows: denaturation at 95 °C for 30 s, annealing at 60 °C for 45 s, and extension at 72 °C for 30 s, and final extension at 72 °C for 10 min (GeneAmp 9700; Applied Biosystems; Thermo Fisher Scientific, Inc. Waltham, MA, USA). Each PCR product was electrophoresed on a 2% agarose gel stained with ethidium bromide (Sigma-Aldrich, St. Louis, MO, USA) to verify the presence of the expected product. The PCR-amplified products of the mutation-containing region of the *AR* gene were prepared using the BigDye™ Terminator Cycle Sequencing kit (Thermo Fisher Scientific, Inc. Waltham, MA, USA), and results were evaluated using SeqScape software 3.

### 2.7. Bioinformatics Analysis

Primers were controlled at Primer designing tool (https://www.ncbi.nlm.nih.gov/tools/primer-blast/) (accessed on: 18 September 2023). The novelty of the variant and its association with AIS were investigated in ClinVar (https://www.ncbi.nlm.nih.gov/clinvar/) (accessed on: 4 January 2024), Varsome, Human Genomics Community (https://varsome.com/) (accessed on: 4 January 2024), Human Gene Mutation Database (HGMD) (https://www.hgmd.cf.ac.uk/ac/index.php) (accessed on: 5 January 2024), Franklin by Genoox (https://franklin.genoox.com/clinical-db/home) (accessed on: 10 January 2024), and the literature.

### 2.8. Evolutionary Conservation Analysis

We used the protein sequences from 13 vertebrate species in the GenBank database (https://www.ncbi.nlm.nih.gov/genbank/) (accessed on: 5 June 2024) to perform the evolutionary conservation analysis of p.Ala749Val mutation. The species were *Rattus norvegicus* (NP_036634), *Mus musculus* (NP_038504.1), *Ovis aries* (NP_001295513), *Myotis brandtii* (XP_014399114.1), *Bos taurus* (NP_001231056), *Equus caballus* (NP_001157363), *Sus scrofa* (NP_999479), *Canis lupus familiaris* (NP_001406230.1), *Loxodonta africana* (XP_003412790), *Octodon degus* (XP_004644568), *Orycteropus afer* (XP_007954476.1), *Cavia porcellus* (XP_063102061.1) and *Homo sapiens* (NP_000035). The ClustalW tool in Molecular Evolutionary Genetics Analysis software (version 4.0) was used for multiple sequence alignment.

### 2.9. Polymorphism Phenotyping v2 (PolyPhen-2) Analysis

Polymorphism Phenotyping v2 (PolyPhen-2) was used for this study, and is a widely used tool for predicting the impact of amino acid substitutions on protein structure and function. The HumDiv model was used to conduct the analysis, which is optimized for distinguishing between damaging and neutral mutations in human disease genes. The protein accession is P10275-1 (Androgen receptor, Homo sapiens) and the mutation analyzed were Alanine to Valine at position 749. The output metrics score are from 0 to 1, which are sensitivity and specificity, respectively.

## 3. Results

### 3.1. Cytogenetics Results

**Figure 2:**
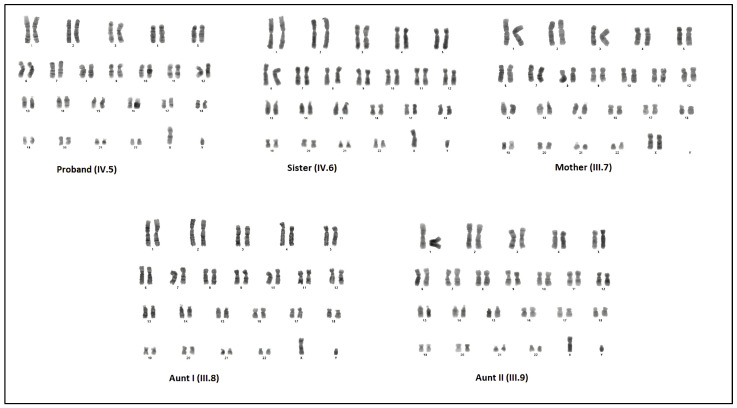
Chromosome analysis results of cases: proband, Sister, Aunt I, and Aunt II with 46,XY karyotype; Mother with 46,XX karyotype.

Chromosome analysis of the proband (IV.5) performed on peripheral blood lymphocytes revealed a 46,XY karyotype with no evidence of mosaicism (50 metaphases examined). The family history suggested X-linked disease. Therefore, it was considered CAIS, and then molecular analysis was performed for the AR gene. A novel hemizygous mutation (c.2246C>T, p.Ala749Val) in the *AR* gene was revealed via NGS analysis. Owing to the presence of atrophic testicles and the increased risk of developing germ cell tumors, bilateral gonad biopsy was recommended in adolescence. Then, a referral was made to the gender committee. Later, chromosome analyses of the Sister (IV.6), Mother (III.7), Aunt I (III.8), and Aunt II (III.9) was also performed from peripheral blood lymphocytes. It was determined that the Sister, Aunt I (III.8), and Aunt II (III.9) had 46,XY karyotype while the Mother (III.7) had normal (46,XX) karyotype. Their karyotypes are shown in Figure 2.

### 3.2. Molecular Results

In this study, we determined a novel hemizygous variant in the *AR* gene (c.2246C>T, p.Ala749Val) of the CAIS patients. This variant was predicted to be pathogenic by Bioinformatics analysis. By analysis of 8 STR markers specific to X and Y chromosomes of 8 STR markers for chromosomes X and Y, it was determined the proband (IV.5), Sister (IV.6), Aunt I (III.8), and Aunt II (III.9) had *SRY* gene, and so they had one X chromosome and one Y chromosome. On the other hand, the Mother (III.7) had no SRY gene, and had two X chromosomes, according to QF-PCR analysis.

### 3.3. Sanger Sequencing Results

**Figure 3:**
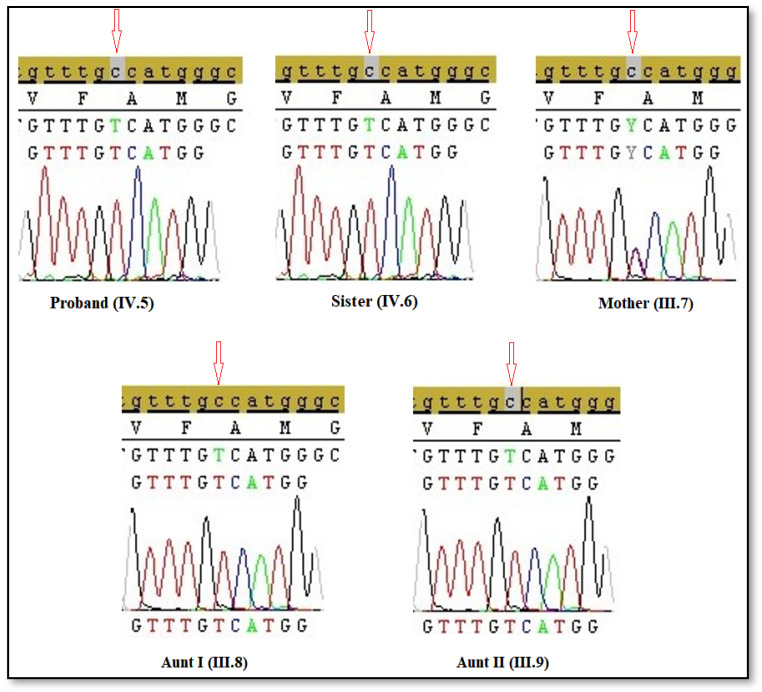
The electropherograms of Sanger sequencing for the five patients.

Sanger sequencing was carried out to verify pathogenic variants in the *AR* gene and to perform genotype/phenotype correlations. Sanger sequencing analysis demonstrated that the proband (IV.5), Sister (IV.6), Aunt I (III.8), and Aunt II (III.9) had hemizygous c.2246C>T mutation, while the Mother (III.7) had heterozygous c.2246C>T mutation. So, it has been thought that this mutation in *AR* gene on X chromosome is of maternal origin. The electropherograms of Sanger sequencing of c.2246C>T mutation in the patients are shown in Figure 3. c.2246C>T mutation is located exon 5 which encodes LBD in *AR* gene.

### 3.4. Evolutionary Conservation Results

**Figure 4:**
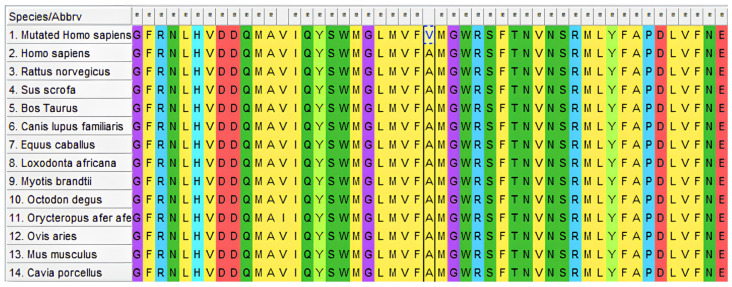
Evolutionary conservation analysis in some vertebrate species for AR.

Evolutionary conservation analysis showed that p.Ala749Val AR mutation in our cases was highly conserved among 13 vertebrate species ranging from *Homo sapiens* to *Cavia porcellus*. Furthermore, exon 5 is completely conserved in this vertebrate species except for p.Ala749Val mutation (Figure 4).

### 3.5. Polymorphism Phenotyping v2 (PolyPhen-2)

**Figure 5:**
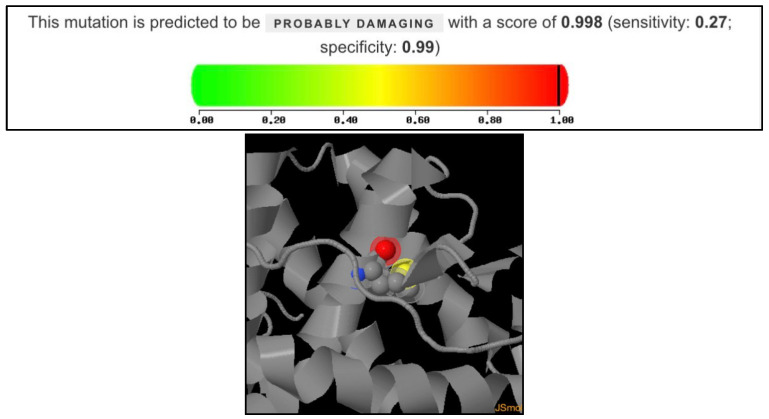
PolyPhen-2 prediction for p.Ala749Val.

The p.Ala749Val variant was predicted by PolyPhen-2 to be probably damaging with a score of 0.998, indicating a high likelihood of detrimental functional consequences. The prediction showed sensitivity: 0.27, specificity: 0.99. This score places the mutation near the upper extreme of PolyPhen-2’s damage prediction scale, suggesting potential structural or functional disruption of the AR protein (Figure 5).

## 4. Discussion

Sex determination in humans is a complex biochemical process that begins during embryonic development, progresses through multiple stages, and continues until adolescence. Numerous genes are involved in regulating this intricate process. AIS is the most common cause of DSD in individuals with a 46,XY karyotype [6]. AIS encompasses a spectrum of phenotypes, ranging from infertile males and individuals with reduced virilization at puberty (PAIS) to those with normal female external genitalia (CAIS) or ambiguous genitalia. This condition is generally attributed to mutations in the AR gene [17,18]. Although an increased tumor risk in patients with CAIS, there is no precise decision when the gonadectomy should be performed. There are some debates in treatment and management of this period. Removal of the gonads to prevent testicular cancer in CAIS is necessity. On the other hand, postponing gonadectomy until at least puberty allows for natural pubertal development delaying the surgery enables the individual to benefit from the physiological effects of puberty, which can be crucial for their overall development and well-being. As a result, there is no definitive decision taken by experts, so each patient should be evaluated individually. However, it is recommended regularly follow patients who do not want to undergo gonadectomy [10]. One of the primary contributing factors to AIS in individuals with a family history of DSD is the presence of inherited mutations. The fundamental cause of AIS is androgen insensitivity due to mutations in the AR gene. Although missense mutations in the *AR* gene can occur in all exons, they are predominantly found in the regions encoding the DNA-binding and ligand-binding domains [19]. Notably, 66.7% of mutations identified in CAIS patients are single-nucleotide missense mutations located within the LBD region [20]. Missense mutations that result in a single amino acid substitution are known to contribute significantly to phenotypic variability [21].

The proband in this study exhibited a completely female external phenotype. Upon further investigation of the proband’s family, five affected individuals were identified. Four of them were diagnosed with CAIS, as they carried the mutation in a hemizygous state. All affected individuals were found to harbor the same *AR* gene mutation (c.2246C>T, p.Ala749Val). Our findings indicate that the hemizygous *AR* gene mutation (p.Ala749Val; c.2246C>T), which has not been previously reported, is consistent with the clinical features observed in the patients. This mutation, located in exon 5 of the AR gene, affects a region that encodes part of the androgen-binding domain and is inherited from the mother. Residue p.Ala749 is located within the *AR* ligand-binding domain, a region essential for hormone recognition and interaction with coactivator proteins. The substitution of alanine, a small nonpolar residue, with valine, a bulkier hydrophobic residue, may cause the local folding environment or disrupt the ligand-binding pocket. The high PolyPhen-2 score supports the hypothesis that the p.Ala749Val mutation could compromise AR functionality, potentially leading to reduced androgen sensitivity or altered receptor signaling.

When reviewing the limited number of studies available in the literature, we encountered research identifying different mutations. A study examining the clinical, hormonal, and genetic characteristics of 128 Turkish patients with 46,XY DSD diagnosed with AIS identified four novel mutations (c.94G>T, c.330G>C, c.2084C>T, and c.2585_2592delAGCTCCTG), as well as a silent mutation (c.330G>C) associated with *AR* gene alterations [22]. Additionally, in a study analyzing the clinical and laboratory data of 51 Turkish children diagnosed with androgen insensitivity, mutations in the *AR* gene (p.Q58L, p.P392S, p.R609K, p.R775H, p.R856H, p.V890M, p.F892L, and the silent mutation p.A871A) were identified in 11 patients (5 PAIS, 6 CAIS) [23]. Furthermore, a 16-year-old Turkish patient with a 46,XY karyotype, female external genitalia, and primary amenorrhea was reported to have a novel hemizygous frameshift mutation in the *AR* gene (c.1629_1630insA) [11].

Loss-of-function mutations in the androgen receptor gene cause AIS. These X-linked mutations disrupt AR activity, resulting in resistance to androgen hormones. Consequently, despite having functional testes and normal testosterone production, individuals with a 46,XY karyotype may present with incomplete masculinization or infertility [24]. The *AR* gene, located on chromosome Xq11–12, includes eight exons and encodes the AR protein, a member of the nuclear receptor superfamily. The AR protein consists of 920 amino acids, and includes four main functional domains [25]. Although more than 600 AIS-associated mutations have been identified—most located in the hormone-binding domain—there are many cases in which no *AR* mutation has yet been detected [24]. Mutations in the *AR* gene can involve substitutions, translocations, deletions, or insertions. These genetic alterations can lead to various changes in the amino acid sequence of the AR protein, typically categorized as silent mutations, missense mutations, nonsense mutations, or mutations that create a stop codon, each affecting the receptor’s function in distinct ways. Moreover, a recent study discovered a novel nonsense mutation in exon 1 of the *AR* gene at position 927 (c.927T>G). This novel mutation is a single-nucleotide substitution (T to G) mutation [25]. Advances in next-generation sequencing have recently led to the discovery of additional mutations responsible for CAIS. Furthermore, the wide variability in genotype-phenotype correlation has prompted investigations into other potential mechanisms contributing to specific forms of partial androgen insensitivity [24]. Another study recently have showed a rare missense mutation 2170C>T (p.Pro274Ser) in *AR* gene resulting CAIS [26]. In our study, we identified a novel mutation and our finding highlights the importance of genetic analysis in diagnosing AIS and improving the understanding of genotype-phenotype correlations.

These findings not only enhance our understanding of AR-related disorders, but also lay the foundation for future research exploring the functional and clinical implications of *AR* mutations, potentially guiding targeted therapeutic strategies for CAIS.

This study has several limitations that should be acknowledged. First, the sample size was limited to a single family, consisting of only five individuals, which restricts the generalizability of our findings. Second, the absence of long-term clinical follow-up data for the affected individuals limits our understanding of potential phenotypic variability or late-onset complications associated with this novel mutation. Future studies involving larger cohorts and functional analyses are needed to confirm the pathogenicity of this novel *AR* gene variant, and to better understand its clinical implications.

## 5. Conclusions

In this study, we identified a novel hemizygous p.Ala749Val variant in the *AR* gene, aligning with an X-linked recessive inheritance pattern. This previously unreported p.Ala749Val substitution occurs at a highly conserved position across mammalian species, underscoring its potential functional significance. Structural analyses suggest that this mutation may critically alter AR activity, contributing to the pathogenesis of CAIS. The p.Ala749Val mutation in the androgen receptor is predicted to be probably damaging to protein function, based on PolyPhen-2 analysis. Given its location in the LBD and potential structural implications, this variant may have pathogenic significance. Given its novelty, this finding provides valuable insights into the molecular etiology of CAIS, expanding our understanding of AR-related disorders and their underlying genetic mechanisms.

## Data Availability

Original data supporting the findings of this study are available. These data are available upon request from the corresponding author.
